# Pre-Eclampsia Comorbid with HIV Infection Mimics the Release of sVCAM-1, sICAM-1, and sE-Selectin in African Women

**DOI:** 10.3390/ijms26178383

**Published:** 2025-08-28

**Authors:** Samukelisiwe Sibiya, Mbuso H. Mthembu, Shoohana Singh, Thajasvarie Naicker, Nompumelelo P. Mkhwanazi

**Affiliations:** 1 HIV Pathogenesis Programme, Doris Duke Medical Research Institute, College of Health Sciences, University of KwaZulu-Natal, Durban 4041, South Africa; 2 Optics and Imaging Centre, Doris Duke Medical Research Institute, College of Health Sciences, University of KwaZulu-Natal, Durban 4041, South Africa; 214556782@stu.ukzn.ac.za (M.H.M.); singhs5@ukzn.ac.za (S.S.); naickera@ukzn.ac.za (T.N.)

**Keywords:** pre-eclampsia, HIV, sVCAM-1, sICAM-1, sE-selectin

## Abstract

Endothelial activation and cell adhesion molecules (CAMs) are exacerbated in the interaction of HIV infection and pre-eclampsia. This study compares the levels of soluble vascular cell adhesion molecule-1 (sVCAM-1), intercellular adhesion molecule-1 (sICAM-1), and E-selectin (sE-selectin) in HIV-infected normotensive pregnant versus pre-eclamptic women. We investigated the plasma concentration of sVCAM-1, sICAM-1, and sE-selectin in normotensive pregnant women (*n* = 40) and pre-eclamptic women (*n* = 40) using an immunoassay procedure. The concentrations of both sVCAM-1 (*p* < 0.0083) and sE-selectin (*p* < 0.0260) were significantly different from sICAM-1 in pre-eclampsia compared to normotensive pregnant groups, irrespective of HIV status. In contrast to sVCAM-1, sICAM-1 (*p* = 0.0349) and sE-selectin (*p* < 0.0445) concentrations were significantly elevated in HIV-positive compared to HIV-negative groups, regardless of pregnancy type. In pregnancies complicated by HIV, statistically significant differences in ICAM-1 concentration were observed between pre-eclamptic HIV-positive versus pre-eclamptic HIV-negative groups (*p* < 0.0010). Similarly, sVCAM-1 levels differed significantly between pre-eclamptic HIV-negative and normotensive HIV-positive groups (*p* < 0.0139). In contrast, sE-selectin levels varied significantly between pre-eclamptic HIV-positive versus normotensive HIV-negative groups (*p* < 0.0485). We report a dysregulation of sICAM-1, sVCAM-1, and SE-selectin in the co-morbidity of pre-eclampsia in pregnant women living with HIV. This differential expression may be attributed to oxidative stress emanating from the hypoxic endothelial activation in both pre-eclampsia and HIV infection and exacerbated by the immune restorative action of antiretroviral therapy.

## 1. Introduction

Pre-eclampsia originates in the placenta, starting with inadequate extravillous cytotrophoblast invasion, and culminates in systemic maternal endothelial dysfunction [1]. Notably, in South Africa (SA), hypertensive disorders in pregnancy (HDP) are the principal direct cause of maternal morbidity and mortality and account for 18% of deaths [2,3]. Moreover, in SA the national HIV antenatal prevalence is high (30–40%) [4,5].

Endothelial cells have an essential homeostatic role in the conservation of vascular tone regulation, and immunomodulation [6]. CAMs of the immunoglobulin (Ig) superfamily, intercellular adhesion molecule 1 (ICAM-1), and vascular cell adhesion molecule 1 (VCAM-1), play a crucial role in leucocyte adherence to endothelial cells during inflammation [7]. During HIV infection, endothelial cells are activated resulting in the release of vascular adhesion molecules and angiogenic factors. This may occur via direct HIV entry into endothelial cells, or indirectly via the release of cytokines, or HIV accessory proteins such as Tat and gp120 on endothelial cells [8]. Consequently, this causes endothelial injury that leads to endothelial dysfunction, predisposed to cardiovascular diseases, especially in patients receiving anti-retroviral therapy (ART) [9,10]. Also, the Tat protein has an arginine–lysine rich sequence analogous to vascular endothelial growth factor (VEGF), fibroblast growth factor, hepatocyte growth factor, and heparin-binding epidermal growth factor, and hence would affect endothelial function and angiogenesis [11].

Pre-eclampsia is also associated with widespread endothelial dysfunction [12,13]. In pre-eclampsia, EC activation and injury predispose a systemic inflammatory response that leads to occlusive thrombotic events mediated by leucocyte recruitment, platelet adhesion and aggregation, blood clotting activation, and fibrinolysis derangement [14]. During leucocyte recruitment, VCAM-1 regulates cell adhesion whilst intercellular adhesion is also influenced by ICAM-1 and E-selectin [15,16]. Of note, the systemic release of soluble forms of these cytokines affects endothelial function [17,18].

Endothelial dysfunction is defined by a shift in endothelium function that precipitates vasoconstriction, inflammation, and prothrombotic properties [19]. This shift emanates from a reduction in nitric oxide (NO) synthesis, oxidative stress, and reduced production of hyperpolarizing factors [20]. This heightened oxidative stress and lipid peroxidation coupled with a deficiency of antioxidant protection contribute to the pathophysiology of endothelial injury [21]. Of note, it is widely documented that the endothelium of human umbilical veins enables direct HIV entry thereby facilitating its infectivity, amplification, and dissemination [22].

Considering the effect of the concomitant EC activation that occurs in pre-eclampsia and HIV infection, one must examine the concentration of these adhesion molecules in the comorbidity of HIV-infected normotensive pregnant versus pre-eclamptic women, receiving ART.

## 2. Results

### 2.1. Patient Demographic and Clinical Characteristics

Patient demographics and clinical characteristics, including gestational age, blood pressure, BMI, and baby weight, are summarized in Table 1. The data is presented as median and IQR. Analysis revealed significant differences across the study groups for gestational age (*p* < 0.0001), systolic blood pressure (*p* < 0.0001), diastolic blood pressure (*p* < 0.0001), BMI (*p* < 0.045), and baby weight (*p* < 0.001). In contrast, no statistically significant differences were observed for maternal age (*p* = 0.2735) or maternal weight (*p* = 0.6618) across the study groups.

### 2.2. Plasma Concentration of Adhesion Markers

The plasma concentrations (pg/mL) of sICAM-1, sVCAM-1, and sE-selectin across all study groups are shown in Table 2 (*n* = 80).

#### 2.2.1. sICAM-1

**Figure 1:**
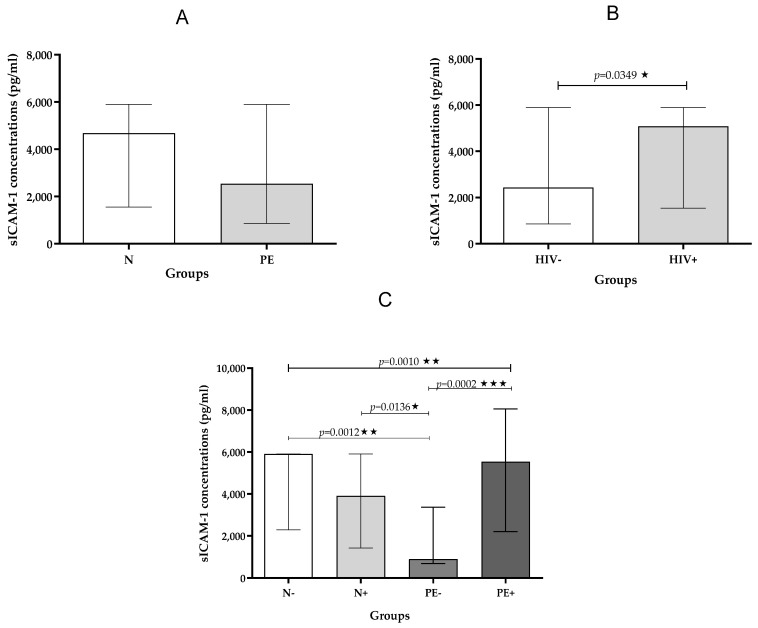
(**A**–**C**): Plasma sICAM-1 concentration stratified by (**A**)—pregnancy type; (**B**)—HIV status and (**C**)—across study groups. Data is presented as the median (IQR); * *p* < 0.05; ** *p* < 0.01, *** *p* < 0.001.

Pregnancy Type: sICAM-1 levels showed no significant difference between pre-eclamptic and normotensive pregnancies, regardless of HIV status, although a slight elevation was observed in the pre-eclamptic group (Table 3; Figure 1A).

HIV Status: In contrast, sICAM-1 concentrations were significantly higher in HIV-positive individuals compared to HIV-negative individuals, irrespective of pregnancy status (*p* = 0.0349; Table 4; Figure 1B).

Across All Groups: When comparing all groups, a significant difference in sICAM-1 levels was found across the groups (Table 2; *p* = 0.0010) with significant decrease in sICAM-1 levels in pre-eclamptic HIV-negative compared to normotensive HIV-negative individuals (*p* = 0.0012). Also noted was a significant decrease in sICAM-1 levels in pre-eclamptic HIV-negative compared to the pre-eclamptic HIV-positive individuals (*p* = 0.0002). Furthermore, there was a significant decrease in sICAM-1 levels in pre-eclamptic HIV-negative compared to the normotensive HIV-positive individuals; (*p* = 0.0136; Figure 1C).

#### 2.2.2. sVCAM-1

**Figure 2:**
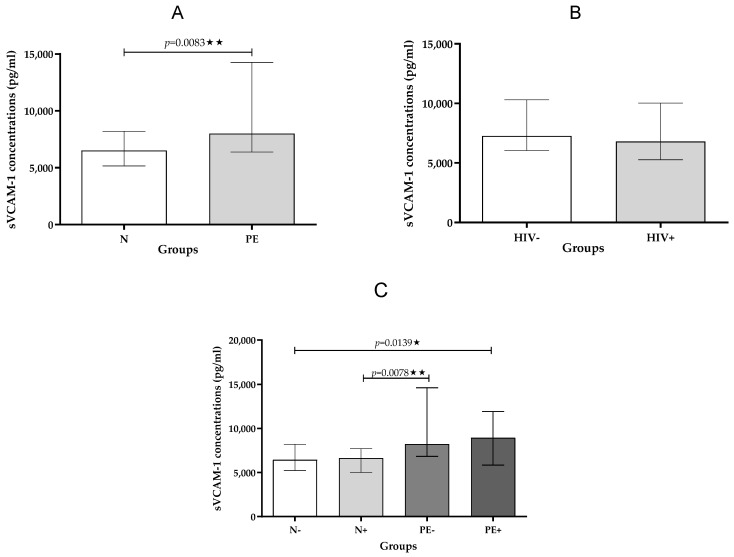
(**A**–**C**): Plasma sVCAM-1 concentration stratified by (**A**)—pregnancy type; (**B**)—HIV status and (**C**)—across study groups. Data is presented as the median (IQR); * *p* < 0.05; ** *p* < 0.01.

Pregnancy Type: Analysis revealed significantly higher sVCAM-1 concentrations in pre-eclamptic pregnancies compared to normotensive pregnancies, regardless of HIV infection status (*p* = 0.0083; Table 3; Figure 2A).

HIV status: In contrast, no significant differences in sVCAM-1 levels were observed between HIV-positive and HIV-negative individuals, irrespective of whether they had pre-eclampsia or not (Table 4; Figure 2B).

Across All Groups: When comparing all groups, a statistically significant difference in sVCAM-1 levels was found across all groups (*p* = 0.0139, Table 2; Figure 2C). Furthermore, a significant increase in sVCAM-1 levels was noted for pre-eclamptic HIV-negative individuals compared to normotensive HIV-positive individuals (*p* = 0.0078).

#### 2.2.3. sE-Selectin

**Figure 3:**
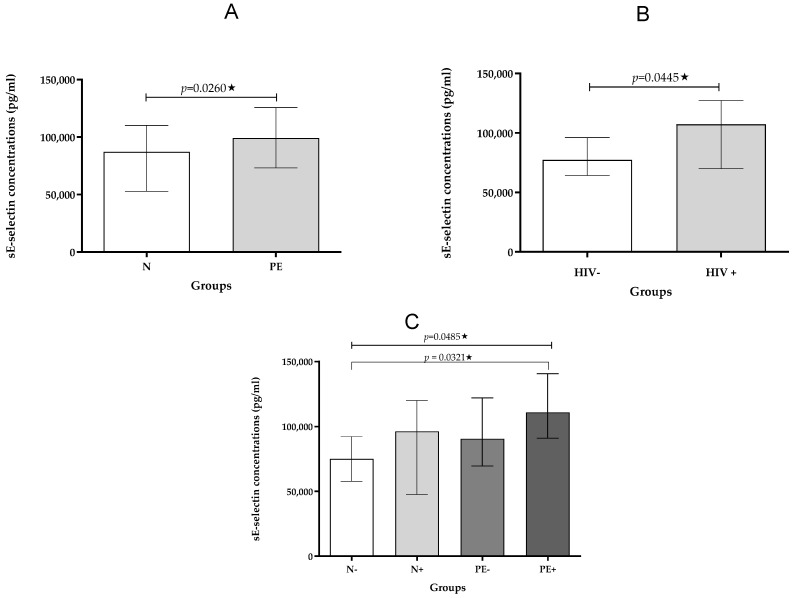
(**A**–**C**): Plasma sE-selectin concentration stratified by (**A**) pregnancy type; (**B**) HIV status, and (**C**) across study groups. Data is presented as the median (IQR); * *p* < 0.05.

Pregnancy Type: Analysis revealed significantly higher sE-selectin concentrations in pre-eclamptic pregnancies compared to normotensive pregnancies, regardless of HIV infection status (*p* < 0.0260; Table 3; Figure 3C).

HIV Status: Furthermore, sE-selectin levels were significantly elevated in HIV-positive individuals compared to HIV-negative individuals, irrespective of whether they had pre-eclampsia or not (*p* = 0.0445; Table 4; Figure 3B).

Across All Groups: When comparing all groups, a statistically significant difference in sE-selectin levels was found across the groups (*p* = 0.0485; Table 2; Figure 3C). A significant increase in sE-selectin levels in pre-eclamptic HIV-positive compared to the normotensive HIV-negative individuals was noted (*p* = 0.0321; Figure 3C).

### 2.3. Correlation Analysis

Gestational age and baby weight were correlated with the cell adhesion markers (Table 5). A moderate negative correlation between sICAM-1 and baby weight in the pre-eclamptic group (r = −0.3912, *p* = 0.0395) was noted. In HIV-positive women, sVCAM-1 showed moderate positive correlations with both gestational age (r = 0.3973, *p* = 0.0221) and baby weight (r = 0.4106, *p* = 0.0269). In HIV-negative women, sE-selectin was positively correlated with gestational age (r = 0.4121, *p* = 0.0293).

## 3. Discussion

The main findings of our study were a significant up-regulation of plasma sVCAM-1 and sE-selectin concentration by pregnancy type (pre-eclampsia compared to normotensive), regardless of HIV status. These results are corroborated by Krauss et al. who reported significantly higher sVCAM-1 levels in the plasma of women diagnosed with EOPE [23]. Notably, our sample population also reflects the EOPE sub-type. It is widely accepted that EOPE is associated with deficient placentation which may be attributed to an elevation of sVCAM-1 and sE-selectin [24]. The deficient trophoblast invasion has been associated with high plasma levels of sICAM-1, sVCAM-1, and sE-selectin and with fetal endothelial dysfunction estimated by low histamine-stimulated nitric oxide (NO) synthesis in pre-eclampsia [25]. Most previous studies have reported elevated cell adhesion molecules albeit in serum rather than plasma of women with pre-eclampsia [17,26]. More recently maternal, fetal, and placental expressions of selectins were demonstrated in pre-eclampsia and correlated with angiotensin receptors (AT1R and AT2R) [27].

Our study also reports a significant elevation of sICAM-1 and sE-selectin in HIV-positive compared to HIV-negative pregnant women, irrespective of pregnancy type (HIV-positive vs. HIV-negative). Notably, an elevation of circulating cell adhesion molecules has been reported in the serum of immune-mediated diseases such as HIV infection which targets immune cells [28]. The HIV regulatory protein, transcriptional trans-activator, *Tat* up-regulates E-selectin expression on endothelial cells whilst its biologically active form, sE-selectin prevents leukocyte adhesion and activates polymorphonuclear cells via the CD11b integrin receptor [28,29]. It is also plausible that the elevated levels of cell adhesion molecules may echo the stimulation of a non-adaptive immune response to HIV infection [30]. More specifically, the virion is believed to increase oxidative stress that triggers cell transduction pathways which promote the release of cell adhesion molecules from activated endothelial cells [31].

Our correlation analysis showed that there were a number of statistically significant links between cell adhesion molecules and clinical parameters. Notably, in the pre-eclamptic group, a moderate negative correlation was observed between sICAM-1 and birthweight (r = −0.3912, *p* = 0.0395), suggesting that heightened endothelial activation may contribute to reduced fetal growth, consistent with the known pathophysiology of early-onset pre-eclampsia. In HIV-positive pregnancies, sVCAM-1 showed a moderate positive correlation with both gestational age (r = 0.3973, *p* = 0.0221) and baby weight (r = 0.4106, *p* = 0.0269). This could reflect a compensatory or adaptive endothelial response as pregnancy progresses or varying effects of antiretroviral therapy on placental function and fetal development. Additionally, in HIV-negative women, sE-selectin positively correlated with gestational age (r = 0.4121, *p* = 0.0293), potentially indicating a physiological increase in endothelial activation with advancing gestation. These findings highlight distinct patterns of endothelial response influenced by both disease state and HIV infection, suggesting that gestational age and birthweight may be differentially affected by the endothelial environment in these clinical contexts.

Pre-eclampsia is also associated with endothelial stress mediated by a hypoxic environment that causes oxidative stress. The binding of HIV-1 to ICAM-1 and VCAM-1 via acquired integrins lymphocyte function-associated antigen 1 (LFA-1) and very late activation antigen 4 (VLA-4) increases its infectivity thereby promoting its dissemination [32]. Both LFA-1 and VLA-4 are considered adhesion molecules as they mediate the attachment of leukocytes to endothelial cells, a crucial step in the inflammatory process [33]. Studies have shown that LFA-1 contributes significantly to HIV infection. It is a key factor in the initial binding of the virus to immune cells and thus enhances viral dissemination and potentially immune dysregulation and consequently their synergistic effects on endothelial function [34,35]. Thus, our findings of elevated CAMs may have a profound impact on HIV infectivity.

Despite ARTs increasing the survival of HIV-infected persons, they are associated with some differential effects on endothelial cell activation and inflammation [36]. Whilst HAART directly induces immune restoration, it also activates endothelial cell injury [37]. Moreover, this metabolic dysregulation involves lipid alteration and insulin resistance which exacerbate endothelial cell activation thereby promoting their injury [38].

The antiretroviral regimen of zidovudine, efavirenz, and either indinavir or nelfinavir can increase mononuclear cell adhesion and ICAM-1 gene expression [39]. This effect is further amplified by the presence of TNF-α, leading to increased expression of ICAM-1, VCAM-1, and endothelial-leukocyte adhesion molecules on the surface of human aortic endothelial cells [40]. Protease inhibitors regulate proteolysis and directly affect cell adhesion leading to neuronal plasticity and synaptic re-organization in the brain that pre-empts learning and memory changes [41]. Protease inhibitors such as ritonavir cause endothelial cell mitochondrial DNA damage and necrosis rather than apoptosis [42]. In contrast, integrase inhibitors down-regulate inflammation to a greater extent than non-nucleoside reverse transcriptase inhibitors (NNRTIs) [43]. It is plausible that the use of nucleoside reverse transcriptase inhibitors (NRTIs) activates the release of reactive oxygen species which leads to elevation of cell adhesion molecules and endothelial cell injury [44]. Notably, NNRTIs such as efavirenz cause a compromise of endothelial cell permeability via intracellular reactive oxygen species (ROS) formation and loss of mitochondrial membrane potential [39]. During short-term acute viremia, there is a rapid and long-lasting endothelial stress response linked to sICAM-1 and sVCAM-1 related to an increased risk of cardiovascular disease overall [45]. Pre-eclampsia is known to increase a woman’s risk of developing cardiovascular disease in later life [46].

In the comorbidity of HIV-infected pregnancies, we report that the concentration of plasma ICAM-1, sVCAM-1, and sE-selectin were significantly different between the pre-eclamptic HIV-positive vs. pre-eclamptic HIV-negative groups (*p* = 0.0002), pre-eclamptic HIV-negative vs. normotensive HIV-positive (*p* = 0.0078) and between the pre-eclamptic HIV-positive vs. normotensive HIV-negative groups (*p =* 0.0321), respectively. The combined effect of endothelial cells dysfunction provoked by its injury in the duality of infection and heightened endothelial cells activation in pre-eclampsia may be further aggravated using ART. Notably, the adhesiveness of endothelial cells represents a balance between activation, proliferation, and cell death [47]. It is also plausible that the up-regulation of soluble adhesion molecules sICAM-1, sVCAM-1, and sE-selectin-1 is a direct result of autoreactive cell degeneration by the restored autoimmune system via the re-constitutive action of ART [48].

We also report a significant reduction in birthweight in the pre-eclamptic HIV-infected groups compared to other study groups. Notably, EOPE is associated with small-for-gestational-age infants due to abnormal trophoblast invasion, with resultant hypoxia emanating from the non-physiological conversion of spiral arteries [49]. In a previous report, ref. [50] observed elevated serum sE-selectin levels in the pre-eclamptic HIV-negative group compared to the normotensive HIV-negative group [50]. However, by pregnancy type no significant difference in sE-selectin was noted; however, this may be attributed to their heterogeneous study population of both early- and late-onset pre-eclampsia. In our study, we utilized a homogenous EOPE group only.

The limitations of our study were the small sample size and the lack of information about the duration of ART. We are unable to report on whether ART was administered at onset, prior to, or during gestation. More recently, HIV-infected individuals on long-term ART show high levels of E-selectin [50]. Viral load, smoking, and other comorbid conditions may influence endothelial activation and cell adhesion molecules [36].

## 4. Materials and Methods

### 4.1. Ethics Approval

Institutional ethics approval (BREC/00002567/2021) was obtained for the use of the retrospectively collected samples (BCA 338/17). In the primary study, the hospital manager’s approval and the Department of Health’s consent to conduct the study was obtained. The study was conducted at a large regional hospital in eThekwini, KwaZulu-Natal, SA. Informed consent was obtained from all participants. Participants’ confidentiality was ensured and remained anonymous.

### 4.2. Study Population

Post consultation with an institutional biostatistician, the sample size was calculated. The study population (*n* = 80) was stratified by pregnancy type into pre-eclamptic (PE; *n* = 40) and normotensive pregnant (N; *n* = 40) women and each group was further sub-divided into HIV-positive (N+; PE+) and HIV-negative (N−; PE−) groups. Inclusion criteria included primigravid and multigravid women diagnosed with pre-eclampsia (≥$\frac{140}{90}$ mmHg) and a minimum of one proteinuria incidence was included [51]. To maintain a homogenous PE group, only those presenting with hypertension before 34 weeks of gestation were included; defined as early onset PE (EOPE). The control group consisted of primigravid and multigravid normotensive pregnant women. HIV status was determined by a rapid test and a CD4+ T cell count was performed (<500 cells/mm^3^). Regardless of CD4+ T cell count, all women received standard HIV treatment during pregnancy and nursing, with continuation of ART for women with CD4+ T cell levels < 350 after breastfeeding. Women received ART either as a single medication, such as zidovudine, commonly known as azidothymidine (AZT), or as a combination of drugs, including tenofovir disoprovil fumarate (TDF, Viread), emtricitabine (FTC, Emtriva), and efavirenz (EFV). Abacavir (ABC, Ziagen), Lamivudine (3TC, Epivir), Efavirenz (EFV), and PMTC (nevirapine) were the alternate drug combinations given to some of the women in accordance with South African National HIV guidelines.

### 4.3. Exclusion Criteria

Women were prohibited from engaging in the study if they refused to give their consent. Polycystic ovary syndrome, chorioamnionitis, eclampsia, chronic hypertension, intrauterine death, abruptio placentae, gestational diabetes, chronic diabetes, systemic lupus erythematosus, chronic renal disease, sickle cell disease, thyroid disease, antiphospholipid antibody syndrome, cardiac disease, pre-existing seizure disorders, and active asthma that requires medication during pregnancy were among the conditions that excluded women from the PE group. Women who were not hospitalized or whose HIV status was unclear were not included.

### 4.4. Sample Collection

The primary trial involved collecting blood samples from eligible women in vacutainer tubes coated with EDTA and centrifuging them at 1000× *g* for 10 min at 4 °C. Ali-quoted plasma was kept in an ultra-freezer at −80 °C until it was needed for analysis.

### 4.5. Bio-Plex Immunoassays

To determine the plasma concentrations of sVCAM-1, sICAM-1, and sE-selectin, a Bio-Plex multiplex immunoassay was performed according to the manufacturer’s guidelines. This assay uses a cytometric mechanism that captures antibody-coupled beads in a 96-well plate.

KIT 1: A Milliplex^®^ MAP Human Cardiovascular 2 MAG (Panel 2) kit was used according to the manufacturer’s instructions (Millipore Sigma Aldrich, Darmstadt, Germany, catalogue number: HCVD2MAG-67K-02). The detection limits for sVCAM-1 and sICAM-1 were, respectively, 0.019 and 0.024 (ng/mL).

KIT 2: A Milliplex^®^ MAP Human Angiogenesis (Panel 2) kit was used according to the manufacturer’s instructions (Millipore Sigma Aldrich, Darmstadt, Germany, catalogue number: HANG2MAG-12K-01). The detection limit for sE-selectin is 116.6 (pg/mL).

The samples (1:40,000 dilutions), standards (serial dilutions), and antibody-immobilized magnetic beads were all incubated in both kits. To make sure that any unbound substances were removed, the plates were then cleaned three times using the wash buffer. After that, the plate was filled with biotinylated detection antibody and left to incubate. The plate was cleaned to get rid of the biotinylated detection antibody after incubation. Each well was filled with streptavidin–phycoerythrin (SA-PE), which was then resuspended in assay buffer after being incubated and cleaned. A Bio-Rad Bio-Plex Magpix system was used for detection. Raw data was extrapolated using Bio-Plex Manager software version 4.1 and the Bio-Plex^®^ MAGPIXTM Multiplex Reader (Bio-Rad Laboratories Inc., Hercules, CA, USA).

### 4.6. Statistical Analysis

Data were analyzed using GraphPad Prism version 5.00 for Windows (GraphPad Software, San Diego, CA, USA). The Kolmogorov–Smirnov test was used to assess the distribution of the data. Owing to non-parametric distributions, results are presented as medians with interquartile ranges (IQRs). Group comparisons by pregnancy type (normotensive vs. pre-eclamptic) and HIV status (negative vs. positive) were performed using the Mann–Whitney U test. For multiple group comparisons (normotensive HIV-negative, normotensive HIV-positive, pre-eclamptic HIV-negative, and pre-eclamptic HIV-positive), a Kruskal–Wallis test was used, followed by post hoc correction for multiple comparisons using the Benjamini–Hochberg false discovery rate (FDR) procedure. Correlations between gestational age or baby birthweight and cell adhesion molecule levels were assessed using Spearman’s rank correlation coefficient. Statistical significance was considered at *p* < 0.05.

## 5. Conclusions

Our findings demonstrate significant differences in plasma concentrations of sVCAM-1 and sE-selectin, but not sICAM-1, in pre-eclamptic pregnancies compared to normotensive pregnancies, regardless of HIV status. This observation may be linked to the heightened inflammatory state and hypoxia associated with defective placentation characteristic of pre-eclampsia.

Furthermore, we observed significantly elevated sICAM-1 and sE-selectin concentrations in HIV-positive compared to HIV-negative individuals, irrespective of pregnancy status. This finding suggests a potential role for ART in modulating the immune response, possibly via the immune reconstitution process.

We report that the comorbidity of HIV-infected pregnancies had significantly different plasma ICAM-1, sVCAM-1, and sE-selectin concentrations. The combination of increased endothelial cell activation in pre-eclampsia and endothelial cell dysfunction brought on by its damage in the setting of HIV is aggravated by ART usage.

## Figures and Tables

**Table 1:** Patient demographics across study groups (*n* = 80).

	Normotensive HIV-Negative	Normotensive HIV-Positive	Pre-Eclampsia HIV-Negative	Pre-Eclampsia HIV-Positive	*p*-Value
Maternal Age (years)	23.00 (19.00–27.00)	28.00 (23.00–35.00)	28.00 (23.00–37.00)	29.00 (26.00–30.50)	0.2735
Gestational Age (weeks)	27.00 (39.00–40.00)	27.00 (37.00–40.00)	24.00 (27.00–23.00)	23.00 (26.50–22.00)	0.0001 ***
Systolic BP (mmHg)	124 (114.5–127.5)	115 (106.5–121.0)	162 (123.0–173.5)	165 (155.5–177.0)	0.0001 ***
Diastolic BP (mmHg)	75.00 (65.50–86.00)	70.00 (67.50–75.00)	98.00 (78.00–110.0)	106.0 (96.50–117.5)	0.0001 ***
Weight (kg)	75.00 (70.50–86.90)	75.00 (68.00–80.25)	68.50 (63.35–83.55)	72.00 (65.55–85.00)	0.6618
BMI (kg/m^2^)	30.54 (27.62–34.00)	27.69 (24.29–31.62)	28.53 (24.27–32.16)	34.30 (25.50–37.44))	0.045 *
Baby weight (kg)	3.20 (3.02–3.76)	3.34 (2.90–3.49)	2.53 (1.50–2.80)	2.60 (1.80–2.86)	0.001 **

Patients’ demographics amongst study group (*n* = 80). Results are presented as the median (IQR); * *p* < 0.05, ** *p <* 0.001, and *** *p* < 0.0001.

**Table 2:** Plasma concentrations (pg/mL) of sICAM-1, sVCAM-1, and sE-selectin across all study groups.

	Normotensive HIV-Negative	Normotensive HIV-Positive	Pre-Eclampsia HIV-Negative	Pre-Eclampsia HIV-Positive	*p* Value
sICAM-1 pg/mL	5904 (2290–5904)	3905 (1434–5904)	900.0 (693.2–3374)	5533 (2210–8054)	0.0010 **
sVCAM-1 pg/mL	6443 (5223–8205)	6641 (5507–7790)	8227 (6832–145,610)	8949 (5831–11,915)	0.0139 *
sE-selectin pg/mL	75,039 (57,769–92,196)	96,231 (47,572–119,905)	90,514 (69,581–121,906)	110,685 (91,056–140,562)	0.0485 *

Patients’ plasma concentrations amongst study group (*n* = 80). Results are presented as the median (IQR); * *p* < 0.05; ** *p* < 0.01.

**Table 3:** Plasma concentrations (pg/mL) of sICAM-1, sVCAM-1, and sE-selectin of pre-eclamptic compared to Normotensive groups.

	Normotensive	Pre-Eclampsia	*p* Value
sICAM-1 pg/mL	4685 (1557–5904)	2543 (866–5904)	ns
sVCAM-1 pg/mL	6515 (5162–8205)	8010 (6382–14,269)	0.0083 **
sE-selectin pg/mL	87,133 (52,948–110,126)	99,190 (73,146–125,653)	0.0260 *

Patients’ plasma concentrations amongst study group (*n* = 80). Results are presented as the median (IQR); * *p* < 0.05; ** *p* < 0.01, ns—no significance.

**Table 4:** Plasma concentrations (pg/mL) of sICAM-1, sVCAM-1 and sE-selectin of HIV-negative compared to HIV-positive groups.

	HIV-Negative	HIV-Positive	*p* Value
sICAM-1 pg/mL	2432 (866–5904)	5082 (1537–5904)	0.0349 *
sVCAM-1 pg/mL	7274 (6047–10,302)	6803 (5274–10,031)	ns
sE-selectin pg/mL	77,279 (64,159–96,092)	107,249 (69,949–127,279)	0.0445 *

Patients’ plasma concentrations amongst study group (*n* = 80). Results are presented as the median (IQR); * *p* < 0.05; ns—no significance.

**Table 5:** Correlation of cell adhesion marker with gestational age and baby weight.

	Gestational Age (Weeks)		Baby Weight (kg)	
	*r*	*p*	*r*	*p*
sICAM-1				
Normotensive pregnancy	–0.1546	0.4414	0.2473	0.2136
Pre-eclamptic pregnancy	–0.1652	0.4008	–0.3912	0.0395 *
HIV-positive	0.1664	0.4165	0.0830	0.6996
HIV-negative	–0.0206	0.9169	–0.2789	0.1769
sVCAM-1				
Normotensive pregnancy	0.2655	0.1232	–0.0898	0.6078
Pre-eclampsia	–0.1114	0.5371	–0.1340	0.4572
HIV-positive	0.3973	0.0221 *	0.4106	0.0269 *
HIV-negative	0.1418	0.4239	0.0824	0.6707
sE-selectin				
Normotensive pregnancy	0.3517	0.0613	–0.1198	0.5358
Pre-eclamptic pregnancy	0.0436	0.8253	0.0273	0.8900
HIV-positive	0.0792	0.6944	0.0096	0.9636
HIV-negative	0.4121	0.0293 *	0.2890	0.1612

* *p* < 0.05.

## Data Availability

The data are unavailable due to privacy or ethical restrictions.

## References

[1] Wang A., Rana S., Karumanchi S.A. (2009). Preeclampsia: The role of angiogenic factors in its pathogenesis. Physiology.

[2] Moodley J., Soma-Pillay P., Buchmann E., Pattinson R.C. (2019). Hypertensive disorders in pregnancy: 2019 National guideline. S. Afr. Med. J..

[3] Naidoo N., Moodley J., Naicker T. (2021). Maternal endothelial dysfunction in HIV-associated preeclampsia comorbid with COVID-19: A review. Hypertens. Res..

[4] Woldesenbet S., Kufa T., Manda S., Ayalew K., Lombard C., Cheyip M., Puren A. (2022). Association between viral suppression during the third trimester of pregnancy and unintended pregnancy among women on antiretroviral therapy: Results from the 2019 antenatal HIV Sentinel Survey, South Africa. PLoS ONE.

[5] Clouse K., Malope-Kgokong B., Bor J., Nattey C., Mudau M., Maskew M. (2020). The South African National HIV Pregnancy Cohort: Evaluating continuity of care among women living with HIV. BMC Public Health.

[6] Vallance P., Collier J., Bhagat K. (1997). Infection, inflammation, and infarction: Does acute endothelial dysfunction provide a link?. Lancet.

[7] Reglero-Real N., García-Weber D., Millán J. (2016). Cellular barriers after extravasation: Leukocyte interactions with polarized epithelia in the inflamed tissue. Mediat. Inflamm..

[8] Anand A.R., Rachel G., Parthasarathy D. (2018). HIV proteins and endothelial dysfunction: Implications in cardiovascular disease. Front. Cardiovasc. Med..

[9] Krishnaswamy G., Kelley J., Yerra L., Smith J.K., Chi D.S. (1999). Human endothelium as a source of multifunctional cytokines: Molecular regulation and possible role in human disease. J. Interferon Cytokine Res..

[10] Xu S., Ilyas I., Little P.J., Li H., Kamato D., Zheng X., Luo S., Li Z., Liu P., Han J. (2021). Endothelial dysfunction in atherosclerotic cardiovascular diseases and beyond: From mechanism to pharmacotherapies. Pharmacol. Rev..

[11] Soga N., Namba N., McAllister S., Cornelius L., Teitelbaum S.L., Dowdy S.F., Kawamura J., Hruska K.A. (2001). Rho family GTPases regulate VEGF-stimulated endothelial cell motility. Exp. Cell Res..

[12] Sánchez-Aranguren L.C., Prada C.E., Riaño-Medina C.E., Lopez M. (2014). Endothelial dysfunction and preeclampsia: Role of oxidative stress. Front. Physiol..

[13] Phipps E.A., Thadhani R., Benzing T., Karumanchi S.A. (2019). Pre-eclampsia: Pathogenesis, novel diagnostics and therapies. Nat. Rev. Nephrol..

[14] Redman C.W., Sargent I.L. (2004). Preeclampsia and the systemic inflammatory response. Seminars in Nephrology.

[15] Singh V., Kaur R., Kumari P., Pasricha C., Singh R. (2023). ICAM-1 and VCAM-1: Gatekeepers in various inflammatory and cardiovascular disorders. Clin. Chim. Acta.

[16] Cook-Mills J.M., Marchese M.E., Abdala-Valencia H. (2011). Vascular cell adhesion molecule-1 expression and signaling during disease: Regulation by reactive oxygen species and antioxidants. Antioxid. Redox Signal..

[17] Kim S.Y., Ryu H.M., Yang J.H., Kim M.Y., Ahn H.K., Lim H.J., Shin J.S., Woo H.J., Park S.Y., Kim Y.M. (2004). Maternal serum levels of VCAM-1, ICAM-1 and E-selectin in preeclampsia. J. Korean Med. Sci..

[18] Kefaloyianni E. (2022). Soluble forms of cytokine and growth factor receptors: Mechanisms of generation and modes of action in the regulation of local and systemic inflammation. FEBS Lett..

[19] Rajendran P., Rengarajan T., Thangavel J., Nishigaki Y., Sakthisekaran D., Sethi G., Nishigaki I. (2013). The vascular endothelium and human diseases. Int. J. Biol. Sci..

[20] Widlansky M.E., Gutterman D.D. (2011). Regulation of endothelial function by mitochondrial reactive oxygen species. Antioxid. Redox Signal..

[21] Mazzuca P., Caruso A., Caccuri F. (2018). Endothelial Cell Dysfunction in HIV-1 Infection. Endothelial Dysfunction: Old Concepts and New Challenges.

[22] Siow R.C. (2012). Culture of human endothelial cells from umbilical veins. Hum. Cell Cult. Protoc..

[23] Krauss T., Kuhn W., Lakoma C., Augustin H.G. (1997). Circulating endothelial cell adhesion molecules as diagnostic markers for the early identification of pregnant women at risk for development of preeclampsia. Am. J. Obstet. Gynecol..

[24] Wojtowicz A., Zembala-Szczerba M., Babczyk D., Kołodziejczyk-Pietruszka M., Lewaczyńska O., Huras H. (2019). Early-and late-onset preeclampsia: A comprehensive cohort study of laboratory and clinical findings according to the new ISHHP criteria. Int. J. Hypertens..

[25] Deer E., Herrock O., Campbell N., Cornelius D., Fitzgerald S., Amaral L.M., LaMarca B. (2023). The role of immune cells and mediators in preeclampsia. Nat. Rev. Nephrol..

[26] Lyall F., Greer I.A. (1994). Pre-eclampsia: A multifaceted vascular disorder of pregnancy. J. Hypertens..

[27] Mistry H.D., Ogalde M.V.H., Broughton Pipkin F., Escher G., Kurlak L.O. (2020). Maternal, Fetal, and Placental Selectins in Women With Pre-eclampsia; Association With the Renin-Angiotensin-System. Front. Med..

[28] Gearing A.J., Newman M. (1993). Circulating adhesion molecules in disease. Immunol. Today.

[29] Lo S.K., Lee S., Ramos R.A., Lobb R., Rosa M., Chi-Rosso G., Wright S.D. (1991). Endothelial-leukocyte adhesion molecule 1 stimulates the adhesive activity of leukocyte integrin CR3 (CD11b/CD18, Mac-1, αmβ2) on human neutrophils. J. Exp. Med..

[30] Harjunpää H., Llort Asens M., Guenther C., Fagerholm S.C. (2019). Cell adhesion molecules and their roles and regulation in the immune and tumor microenvironment. Front. Immunol..

[31] Tzavara V., Vlachoyiannopoulos P.G., Kordossis T., Galaris D., Travlou A., Dafni U., Moutsopoulos H.M. (1997). Evidence for non-adaptive immune response in HIV infection. Eur. J. Clin. Investig..

[32] Aouache R., Biquard L., Vaiman D., Miralles F. (2018). Oxidative stress in preeclampsia and placental diseases. Int. J. Mol. Sci..

[33] Chan P.Y., Aruffo A. (1993). VLA-4 integrin mediates lymphocyte migration on the inducible endothelial cell ligand VCAM-1 and the extracellular matrix ligand fibronectin. J. Biol. Chem..

[34] Liao Z., Roos J.W., Hildreth J.E. (2000). Increased infectivity of HIV type 1 particles bound to cell surface and solid-phase ICAM-1 and VCAM-1 through acquired adhesion molecules LFA-1 and VLA-4. AIDS Res. Hum. Retroviruses.

[35] Card C.M., Abrenica B., McKinnon L.R., Ball T.B., Su R.C. (2022). Endothelial cells promote productive HIV infection of resting CD4+ T cells by an integrin-mediated cell adhesion-dependent mechanism. AIDS Res. Hum. Retroviruses.

[36] Zicari S., Sessa L., Cotugno N., Ruggiero A., Morrocchi E., Concato C., Rocca S., Zangari P., Manno E.C., Palma P. (2019). Immune activation, inflammation, and non-AIDS co-morbidities in HIV-infected patients under long-term ART. Viruses.

[37] Naicker T., Govender N., Abel T., Naidoo N., Moodley M., Pillay Y., Singh S., Khaliq O.P., Moodley J. (2021). HIV associated preeclampsia: A multifactorial appraisal. Int. J. Mol. Sci..

[38] Yang Q., Vijayakumar A., Kahn B.B. (2018). Metabolites as regulators of insulin sensitivity and metabolism. Nat. Rev. Mol. Cell Biol..

[39] Jamaluddin M.S., Lin P.H., Yao Q., Chen C. (2010). Non-nucleoside reverse transcriptase inhibitor efavirenz increases monolayer permeability of human coronary artery endothelial cells. Atherosclerosis.

[40] Mondal D., Pradhan L., Ali M., Agrawal K.C. (2004). HAART Drugs Induce Oxidative Stress in Human Endothelial Cells and Increase Endothelial Recruitment of Mononuclear Cells: Exacerbation by Inflammatory Cytokines and Amelioration by Antioxidants. Cardiovasc Toxicol..

[41] Lee T.W., Tsang V.W., Birch N.P. (2008). Synaptic plasticity-associated proteases and protease inhibitors in the brain linked to the processing of extracellular matrix and cell adhesion molecules. Neuron Glia Biol..

[42] Zong D., Lu X., Conklin B.S., Lin P.H., Lumsden A.B., Yao Q., Chen C. (2002). HIV protease inhibitor ritonavir induces cytotoxicity of human endothelial cells. Arterioscler. Thromb. Vasc. Biol..

[43] Hileman C.O., Funderburg N.T. (2017). Inflammation, immune activation, and antiretroviral therapy in HIV. Curr. HIV/AIDS Rep..

[44] Mitchell D., Israr M., Alam S., Dinello D., Kishel J., Jia R., Meyers C. (2014). HIV nucleoside reverse transcriptase inhibitors efavirenz and tenofovir change the growth and differentiation of primary gingival epithelium. HIV Med..

[45] Papasavvas E., Azzoni L., Pistilli M., Hancock A., Reynolds G., Gallo C., Ondercin J., Kostman J.R., Mounzer K., Shull J. (2008). Increased soluble vascular cell adhesion molecule-1 plasma levels and soluble intercellular adhesion molecule-1 during antiretroviral therapy interruption and retention of elevated soluble vascular cellular adhesion molecule-1 levels following resumption of antiretroviral therapy. AIDS.

[46] Conti-Ramsden F., Bramham K., de Marvao A. (2024). Long-term cardiovascular disease after pre-eclampsia: Time to move from epidemiology to action. Eur. Heart J.-Qual. Care Clin. Outcomes.

[47] Francisci D., Giannini S., Baldelli F., Leone M., Belfiori B., Guglielmini G., Malincarne L., Gresele P. (2009). HIV type 1 infection, and not short-term HAART, induces endothelial dysfunction. AIDS.

[48] Matarese G., De Placido G., Nikas Y., Alviggi C. (2003). Pathogenesis of endometriosis: Natural immunity dysfunction or autoimmune disease?. Trends Mol. Med..

[49] Odukoya S.A., Moodley J., Naicker T. (2021). Current Updates on Pre-eclampsia: Maternal and Foetal Cardiovascular Diseases Predilection, Science or Myth? Future cardiovascular disease risks in mother and child following pre-eclampsia. Curr. Hypertens. Rep..

[50] Hoffman M., Ipp H., Phatlhane D.V., Erasmus R.T., Zemlin A.E. (2018). E-Selectin and markers of HIV disease severity, inflammation and coagulation in HIV-infected treatment-naïve individuals. Afr. Health Sci..

[51] Magee L.A., Smith G.N., Bloch C., Côté A.M., Jain V., Nerenberg K., von Dadelszen P., Helewa M., Rey E. (2022). Guideline No. 426: Hypertensive disorders of pregnancy: Diagnosis, prediction, prevention, and management. J. Obstet. Gynaecol. Can..

